# Epidemic of HIV infection among persons who inject drugs in mainland China: a series, cross-sectional study

**DOI:** 10.1186/s12954-021-00511-6

**Published:** 2021-06-12

**Authors:** Bo Zhang, Xiangyu Yan, Yongjie Li, He Zhu, Zhimin Liu, Zuhong Lu, Zhongwei Jia

**Affiliations:** 1 School of Basic Medical Sciences, Peking University, Beijing, 100191 China; 2 School of Public Health, Peking University, Beijing, 100191 China; 3 China Center for Health Development Studies, Peking University, Beijing, 100191 China; 4 National Institute on Drug Dependence, Peking University, Beijing, 100191 China; 5 State Key Laboratory for Bioelectronics, School of Biological Science and Medical Engineering, Southeast University, Nanjing, 211189 China; 6 Center for Intelligent Public Health, Institute for Artificial Intelligence, Peking University, Beijing, 100191 China; 7 Center for Drug Abuse Control and Prevention, National Institute of Health Data Science, Peking University, Beijing, 100191 China

**Keywords:** Epidemic, Persons who inject drugs, HIV, Injection drug use

## Abstract

**Background:**

Studies have suggested that the injection drug use (IDU) was no longer the main transmission route of HIV/AIDS in China. However, there has never been a study to assess the national HIV epidemic among persons who inject drugs (PWIDs) based on a nationwide database.

**Methods:**

PWIDs among new entrants in detoxification centers with HIV test results were extracted from the 2008–2016 National Dynamic Management and Control Database for Persons Who Use Drugs (NDMCD). Logistic regressions were used to analyze factors associated with HIV infection, and joinpoint regression were used to examine trends in the HIV prevalence.

**Results:**

A total of 103,619 PWIDs among new entrants tested for HIV in detoxification centers between 2008 and 2016 were included in the analysis. The HIV prevalence was 5.0% (*n* = 5167) among PWIDs. A U-shaped curve of the HIV prevalence decreased from 4.9% in 2008 to 3.3% in 2010 (Annual Percent Change [APC] − 20.6, 95% CI − 32.5 to − 6.7, *p* < 0.05) and subsequently increased from 3.3% in 2010 to 8.6% in 2016 (APC 17.9, 95% CI 14.5–21.4, *p* < 0.05) was observed. The HIV prevalence in west regions in China all presented decreased trends, while central and eastern regions presented increased trends.

**Conclusions:**

Although the HIV prevalence has been declining in general population, the HIV prevalence among PWIDs has shown an increasing trend since 2010. Current policies on HIV control in PWIDs should be reassessed.

**Supplementary Information:**

The online version contains supplementary material available at 10.1186/s12954-021-00511-6.

## Background

Injection drug use (IDU) is a leading cause of HIV infections because of sharing contaminated injection equipment [1]. Among persons who inject drugs (PWIDs), HIV could be transmitted through sexual contact and injection-related risk behavior, and injection-related transmission is thought to be the dominant route in most settings [2]. According to a recent study covering 99% of the population aged 15–64 years globally, the number of countries reported IDU has increased from 148 in 2008 to 179 in 2017 [3, 4]. Moreover, the global prevalence of HIV among PWIDs was 17.8% in 2017, and PWIDs were estimated to be 36 times more likely than those in the general population to be living with HIV [4, 5].

In China, the first reported 79 HIV cases among PWIDs were in Dehong, Yunnan Province in 1989 [6]. Prior to 2007, IDU were predominantly responsible for new HIV/AIDS infections [7, 8]. However, the proportion of new HIV infections through sexual transmission exceeded IDU and became the primary transmission route in 2007 [8, 9]. Several previous studies using surveillance data also indicated that the national HIV prevalence among PWIDs has decreased slightly from 10.6% in 2002 to 9.1% in 2010 [10, 11]. Lan Wang et al. found that trends in the HIV prevalence in China peaked at 30.3% among PWIDs in 1999, and then gradually decreased to 10.9% in 2011 [12]. Correspondingly, the needle sharing behavior among PWIDs has decreased from 19.5% in 2006 to 11.3% in 2011 [12]. This was made possible by the inclusion of harm reduction strategies, such as opioid substitution treatment (OST) and needle and syringe programs (NSPs) [12]. Lei Zhang et al. systematically analyzed trends in the HIV prevalence by regions and subpopulation groups from 1995 to 2010, and found the prevalence of HIV infection among PWIDs has been decreasing in all regions except for southwest China and has stabilized at a high level in northwest China [13].

National Dynamic Management and Control Database (NDMCD) is a national registry database set up by China government to register persons who use drugs (PWUDs), and it includes all administrative streets and blocks in the 31 provinces over mainland China [14]. NDMCD was designed to establish a management paradigm suitable for effective surveillance of drug use and related diseases in China [14]. Data were collected by government staffs, and drug types used by PWUDs were verified by urine tests, which are more accurate than data from questionnaires of sentinel surveillance sites [14–16]. All PWUDs registered in NDMCD would receive community-based or institute-based help, education and detoxification treatment [16]. Additionally, among PWUDs who need to receive detoxification treatment, HIV testing service is provided before they enter detoxification centers.

Taken together, HIV infections among PWIDs is an essential public health concern in China, and has important implications in HIV control; however, studies of nationwide HIV epidemic among PWIDs in recent years were scarce. Therefore, this study aims to assess the epidemic of HIV among PWIDs in detoxification centers nationwide from 2008 to 2016 by analyzing data extracted from the NDMCD, and we examine the HIV prevalence and its trends in PWIDs.

## Methods

### Definitions

*PWUDs*: Individuals have been found ever used prohibited psychotropic and narcotic drugs for non-medical purposes and registered in the NDMCD.

*PWIDs*: Individuals had self-reported ever IDU behavior and be recorded in the NDMCD.

*New entrants in detoxification centers*: Drug users who entered in detoxification centers were tested HIV for the first time. According to the guideline of “measures for the judgment of drug addiction,” the professional medical workers judged whether PWUDs need to enter the detoxification centers for drug treatment. PWUDs received drug-related diagnosis and evaluation, treatment, rehabilitation training, and educational and psychological correction. PWUDs in the detoxification centers must receive two years’ inpatient treatment.

*HIV prevalence*: HIV prevalence was calculated by the number of HIV-positive individuals (numerator) divided by the number of individuals who had done HIV test (denominator) among new entrants in detoxification centers, stratified by year.

### Study design and study sample

New entrants who were PWIDs in detoxification centers tested for HIV between 2008 and 2016 were included in this analysis. Individuals who had no sociodemographic characteristics data were excluded. A total of 103,623 PWIDs were extracted from NDMCD. We excluded four individuals without sociodemographic characteristics data; finally, 103,619 PWIDs were included in the analysis.

PWUDs received HIV test service before entered detoxification centers. All new entrants in detoxification centers also needed to report IDU behavior and the HIV test registered in NDMCD from Jan 2008 to Aug 2016. We extracted sociodemographic characteristics, HIV test date and region, drug types and methadone treatment status before HIV test from the database.

The diagnosis of HIV infection by serological tests included both the screening test and the confirmation test based on "Diagnostic criteria for HIV/AIDS". HIV-positive PWUDs who meet the Chinese national treatment criteria (WHO stage 3 or 4 disease or CD4 count of 350 cells per μl or less) were referred for treatment with standard three-drug therapy. Primary outcomes were the prevalences and trends in HIV infection among total PWIDs, and secondary outcomes were the prevalences and trends in HIV infection among sub-populations, stratified by socio-demographic characteristics, drug types, HIV-related variables and risk factors related to HIV infection.

### Statistical analysis

Logistic regression models were conducted to calculate unadjusted and adjusted odds ratios (ORs and AORs), respectively, and 95% confidence intervals (CIs) of the HIV prevalence by characteristics [i.e., sex (male and female), age (≤ 24, 25–44, and ≥ 45 years), ethnicity (Han and minority), education (primary school or below, junior high school, and high school or above), marital status (divorced or widowed, married, and unmarried), and methadone treatment status before HIV test (yes or no)]. We also evaluated differences in the HIV prevalence among seven regions of China [northeast (Heilongjiang, Jilin, and Liaoning), north (Inner Mongolia, Shanxi, Hebei, Beijing, and Tianjin), east (Shandong, Jiangsu, Zhejiang, Shanghai, Fujian, Jiangxi, and Anhui), central (Henan, Hubei, and Hunan), south (Guangxi, Guangdong, and Hainan), southwest (Yunnan, Xizang, Sichuan, Chongqing, and Guizhou), and northwest (Xinjiang, Gansu, Ningxia, Qinghai, and Shaanxi)]. Sex, age, ethnicity, education, marital status, and methadone treatment status before HIV test, HIV test year and geographical regions were included into the adjusted analyses.

Joinpoint regression was used to examine the changing trend in the HIV prevalence during the study period. Annual percent change (APC) for each line segment and the corresponding 95% CI were estimated. The APC was tested to determine whether a difference existed from the null hypothesis of no change (0%). Each joinpoint informed a change in trends (increase or decrease) and each of trends was described by an APC.

A two-sided *p* value of 0.05 or less was defined as significant. Original data were double checked in PostgreSQL 9.3 and SAS version 9.4 (SAS Institute, Inc., Cary, NC). Statistical analyses were carried out and verified by using SPSS version 22.0 (IBM Corp) and SAS version 9.4 (SAS INSTITUTE INC). Trend analysis was done with Joinpoint Regression Program 4.6.0. Mapping was done with ArcGIS, version 10.0 (Esri).

## Results

### Demographical characteristics of the study sample

103,619 PWIDs were included in the analysis. In the total sample (*n* = 103,619 PWIDs), 89.3% (*n* = 92,548) were male, median age was 35 years old (IQR: 30–41), 84.7% (*n* = 87,816) was Han ethnicity, 86.6% (*n* = 89,782) had a junior school or below education, 53.0% (*n* = 54,853) were non-marital status, 99.3% (*n* = 102,850) ever used opioids drugs, and 79.6% (*n* = 82,431) never received methadone treatment before entered detoxification centers (Table 1). The residences of all PWIDs included south (30.2%), east (23.1%), central (18.4%), and southwest (16.7%) in China.

**Table 1:** Sociodemographic characteristics, methadone treatment, HIV test year, region, and HIV infections among PWIDs in China, 2008–2016

Characteristics	PWIDs (*N*)	Proportion (%)
Total	103,619	100
Sex		
Male	92,548	89.3
Female	11,071	10.7
Age	35 (30–41)	
Ethnicity		
Han	87,816	84.7
Minority	11,573	11.2
Missing	4230	4.1
Education		
High school or above	11,572	11.2
Junior high school	64,386	62.1
Primary school or below	25,396	24.5
Missing	2265	2.2
Marital status		
Married	47,017	45.4
Unmarried	43,908	42.4
Divorced/widows	10,945	10.6
Missing	1749	1.7
Drug types		
Opioids	102,850	99.3
Other types	769	0.7
Methadone treatment status		
No	82,431	79.6
Yes	21,188	20.4
Year		
2008	8301	8.0
2009	18,206	17.6
2010	14,690	14.2
2011	11,526	11.1
2012	10,905	10.5
2013	12,152	11.7
2014	12,470	12.0
2015	11,092	10.7
2016	4277	4.1
Region		
Southwest	17,318	16.7
Northwest	5702	5.5
South	31,293	30.2
Northeast	1583	1.5
Central	19,032	18.4
North	4752	4.6
East	23,939	23.1
HIV		
Positive	5167	5.0
Negative	98,452	95.0

PWIDs, persons who inject drugs

### HIV prevalence and risk factors

Among 103,619 PWIDs, the HIV prevalence was 5.0% (Table 2). The HIV prevalence among female PWIDs was 5.2%, which was higher than 5.0% among male PWIDs (AOR 1.27, 95% CI 1.15–1.39, *p* < 0.001). Elder age had a higher risk of HIV infection (HIV prevalence 25–44 vs < 25: 5.2% vs 3.1%, AOR 2.31, 95% CI 2.03–2.64, *p* < 0.001; ≥ 45 vs < 25: 5.0% vs 3.1%, AOR 2.57, 95% CI 2.20–3.00, *p* < 0.001). 12.3% of minority PWIDs were HIV positive, and were more likely to be HIV infected compared to 4.1% of Han PWIDs (AOR 2.61, 95% CI 2.43–2.80, *p* < 0.001). Compared with the HIV prevalence (2.9%) of people who had high school or above education, people who had junior school education had a higher risk of HIV infection (AOR 1.41, 95% CI 1.26–1.59, *p* < 0.001), similar to primary school or below education (AOR 2.00, 95% CI 1.77–2.26, *p* < 0.001). Additionally, there was no statistical difference in the HIV prevalence between PWIDs with ever opioids injection and those without opioid injection. Individuals who received methadone treatment before were more likely to be infected HIV (AOR 1.59, 95% CI 1.49–1.71, *p* < 0.001).

**Table 2:** Univariate and multivariable analysis for HIV infection among PWIDs in China, 2008–2016

Characteristics	HIV *n* (%)	Prevalence (%)	OR (95%CI)	AOR (95%CI)
Total	5167 (100.0)	5.0		
Sex				
Male	4589 (88.8)	5.0	1.00	1.00
Female	578 (11.2)	5.2	0.95 (0.87–1.04)	1.27 (1.15–1.39)***
Age				
~ 24	270 (5.2)	3.1	1.00	1.00
25–44	4180 (80.9)	5.2	1.71 (1.51–1.94)***	2.31 (2.03–2.64)***
45 ~	715 (13.8)	5.0	1.64 (1.42–1.89)***	2.57 (2.20–3.00)***
Missing	2 (0.0)	18.2	6.95 (1.49–32.30)*	7.84 (1.48–40.57)*
Ethnicity				
Han	3584 (69.4)	4.1	1.00	1.00
Minority	1419 (27.5)	12.3	3.29 (3.08–3.51)***	2.61 (2.43–2.80)***
Missing	164 (3.2)	3.9	0.95 (0.81–1.11)	0.96 (0.81–1.13)
Education				
High school or above	334 (6.5)	2.9	1.00	1.00
Junior high school	2728 (52.8)	4.2	1.49 (1.33–1.67)***	1.41 (1.26–1.59)***
Primary school or below	1965 (38.0)	7.7	2.82 (2.51–3.18)***	2.00 (1.77–2.26)***
Missing	140 (2.7)	6.2	2.22 (1.81–2.72)***	2.01 (1.36–2.97)***
Marital status				
Married	2106 (40.8)	4.5	1	1
Unmarried	2389 (46.2)	5.4	1.23 (1.16–1.30)***	1.20 (1.13–1.28)***
Divorced/widows	562 (10.9)	5.1	1.15 (1.05–1.27)**	1.08 (0.98–1.20)
Missing	110 (2.1)	6.3	1.43 (1.17–1.75)***	1.25 (0.82–1.92)
Drug types				
Opioids	5139 (99.5)	5.0	1.00	1.00
Other types	28 (0.5)	3.6	0.72 (0.49–1.05)	0.86 (0.58–1.27)
Methadone treatment status				
No	3921 (75.9)	4.8	1.00	1.00
Yes	1246 (24.1)	5.9	1.25 (1.17–1.34)***	1.59 (1.49–1.71)***
Year				
2008	406 (7.9)	4.9	1.00	1.00
2009	775 (15.0)	4.3	0.87 (0.77–0.98)*	0.97 (0.85–1.10)
2010	486 (9.4)	3.3	0.67 (0.58–0.76)***	0.74 (0.64–0.85)***
2011	425 (8.2)	3.7	0.74 (0.65–0.86)***	0.77 (0.67–0.89)***
2012	481 (9.3)	4.4	0.90 (0.78–1.03)	0.82 (0.71–0.94)**
2013	616 (11.9)	5.1	1.04 (0.91–1.18)	0.81 (0.70–0.92)**
2014	748 (14.5)	6.0	1.24 (1.10–1.41)**	0.95 (0.83–1.08)
2015	862 (16.7)	7.8	1.64 (1.45–1.85)***	1.14 (1.00–1.30)*
2016	368 (7.1)	8.6	1.83 (1.58–2.12)***	1.28 (1.09–1.49)**
Region				
Southwest	2285 (44.2)	13.2	1.00	1.00
Northwest	333 (6.4)	5.8	0.41 (0.36–0.46)***	0.42 (0.37–0.47)***
South	1556 (30.1)	5.0	0.34 (0.32–0.37)***	0.39 (0.36–0.41)***
Northeast	128 (2.5)	8.1	0.58 (0.48–0.70)***	0.74 (0.61–0.89)**
Central	321 (6.2)	1.7	0.11 (0.10–0.13)***	0.14 (0.12–0.16)***
North	158 (3.1)	3.3	0.23 (0.19–0.27)***	0.26 (0.22–0.31)***
East	386 (7.5)	1.6	0.11 (0.10–0.12)***	0.13 (0.12–0.15)***

PWIDs, persons who inject drugs

***suggested *p* < 0.001; **suggested *p* < 0.01; *suggested *p* < 0.05

### Trends in the HIV prevalence

**Fig. 1:**
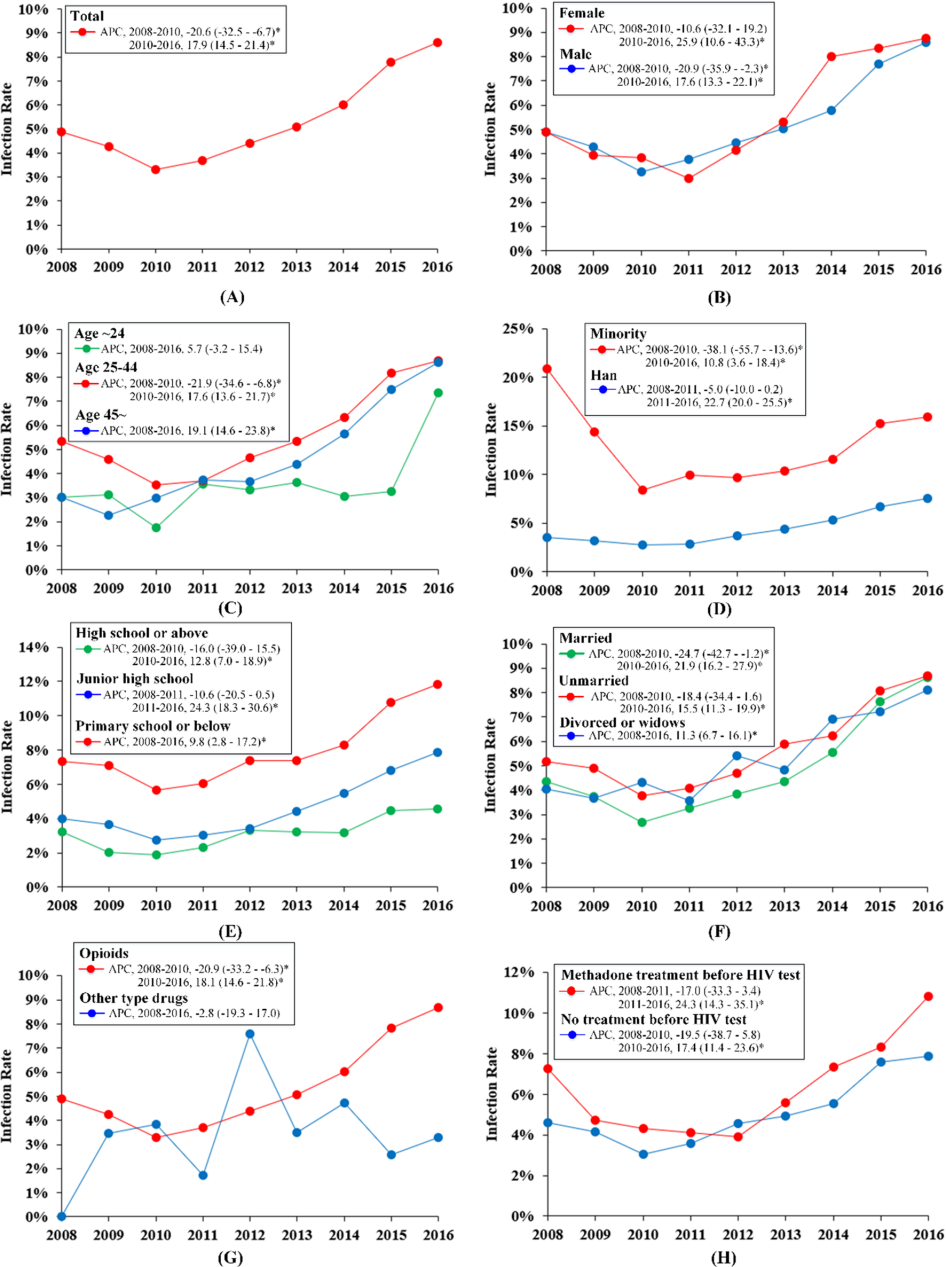
Trends in the prevalence of HIV infection among persons who inject drugs (PWIDs). *suggested *p* < 0.05

A U-shaped curve with 33% decrease (APC − 20.6, 95% CI − 32.5 to − 6.7, *p* < 0.05) in the HIV prevalence from 4.9% in 2008 to 3.3% in 2010 and subsequently 161% increase (APC 17.9, 95% CI 14.5–21.4, *p* < 0.05) from 3.3% in 2010 to 8.6% in 2016 was observed among PWIDs (Table 2, Fig. 1). After adjusted for sociodemographic characteristics, HIV test region, drug types, and methadone treatment status, the U-shaped trend in HIV infections among PWIDs was still observed (Additional file 1: Table S1). Furthermore, subpopulations of HIV infections among PWIDs, such as male [a decrease (APC − 20.9, 95% CI − 35.9 to − 2.3, *p* < 0.05) from 2008 to 2010 and an increase (APC 17.6, 95% CI 13.3–22.1, *p* < 0.05) from 2010 to 2016], young adults (24–45 years old) [a decrease (APC − 21.9, 95% CI − 34.6 to − 6.8, *p* < 0.05) from 2008 to 2010 and an increase (APC 17.6, 95% CI 13.6–21.7, *p* < 0.05) from 2010 to 2016], minority [a decrease (APC − 38.1, 95% CI − 55.7 to − 13.6, *p* < 0.05) from 2008 to 2010 and an increase (APC 10.8, 95% CI 3.6–18.4, *p* < 0.05) from 2010 to 2016], married people [a decrease (APC − 24.7, 95% CI − 42.7 to − 1.2, *p* < 0.05) from 2008 to 2010 and an increase (APC 21.9, 95% CI 16.2–27.9, *p* < 0.05) from 2010 to 2016], and opioids users [a decrease (APC − 20.9, 95% CI − 33.2 to − 6.3, *p* < 0.05) from 2008 to 2010 and an increase (APC 18.1, 95% CI 14.6–21.8, *p* < 0.05) from 2010 to 2016], all showed U-shaped curves for the HIV prevalence. In addition, other subpopulations, such as female, people over 45 years old, Han ethnicity, and non-marital people, all presented increased trends in recent years (Fig. 1).

### Geographical differences in HIV infections

The HIV prevalence in southwest was 13.2%, higher than other regions (Table 2). A decreased trend (APC − 10.0, 95% CI − 15.5 to − 4.1, *p* < 0.05) from 2008 to 2013 was observed in southwest (Additional file 2: Figure S1). Also, the decreased trend (APC − 27.2, 95% CI − 42.2 to − 8.4, *p* < 0.05) was observed in northwest. Yet an increased trend (APC − 27.2, 95% CI − 42.2 to − 8.4, *p* < 0.05) of the HIV prevalence was observed in south from 2011 to 2016. The HIV prevalence in north, central and east regions all presented the increased trends (North: APC 15.7, 95% CI 2.7– 30.4, *p* < 0.05; East: APC 27.6, 95% CI 18.1– 37.8, *p* < 0.05; Central: APC 29.8, 95% CI 20.0– 40.4, *p* < 0.05) from 2008 to 2016 (Additional file 2: Figure S1).

## Discussion

Overall, the HIV prevalence among PWIDs from China’s national data was 5.0%, which was higher than that among the general population (around 0.6‰) [11]. The HIV prevalence among PWIDs presented the U-shaped trend, which decreased from 4.9% in 2008 to 3.3% in 2010 and subsequently increased from 3.3% in 2010 to 8.6% in 2016. Although the HIV prevalence in west regions in China presented decreased trends, it was still the most prevalent area of HIV epidemic.

In China, HIV infection among drug users mainly arose from PWIDs by needle sharing [9, 12]. The WHO recommends that NSPs can effectively reduce HIV transmission among PWIDs, and a meta-analysis also suggested that NSPs were associated with a 58% reduction in HIV transmission [17]. China initiated the first NSP in Yunnan Province in 1998, which gradually has become a nationwide program through the support of The Joint United Nations Program on HIV/AIDS (UNAIDS) and international programs since 2004 [18]. By the end of 2005, over two-thirds of NSP sites were located in southern and western provinces where the HIV among drug users was much more prevalent [19]. And the number of NSP sites increased over years, by 2010, the national government has established about 1000 NSP sites covered at least 50% of PWIDs nationwide [12, 20]. Wei Luo et al. explored the relationship between NSP and HIV infection across China, and found that in 2010, 1928 of 3494 (55.2%) interviewed PWIDs had ever attended NSP at least once; the unadjusted HIV prevalence of NSP non-attendees was 16.5%, which was 1.67 times more likely to be HIV-positive compared to 13.9% of HIV infections among NSP attendees [21]. Due to the expanded NSP coverage in China, the prevalence of HIV among PWIDs have been reduced about one third (from 4.9% in 2008 to 3.3% in 2010) reported by our study.

Although China has scaled up NSPs, it has not been extensively evaluated to explore factors associated with the acceptability and feasibility [22]. In-depth qualitative interviews with 35 PWIDs identified themes including fears of breached confidentiality, police interference in NSP sites, and tensions between the public health and law enforcement perspective [23]. Lei Zhang et al. suggested that continued law enforcement and mandatory detoxification remain as major barriers to the necessary program scale-up that may even counteract the benefits of NSPs [23]. Ongoing police crackdowns, arrests, and confinement substantially discourage PWIDs from contacting peer health educators and accessing to NSP sites [23]. NSP should be expanded to include those who had not yet attended the program before, but there are few relevant data and reports on nationwide NSP after 2010. UNAIDS 2020 data reported a high coverage of needle-syringe programs (246 needles and syringes per person who injects drugs per year) in China, but the coverage might be overestimated because those who had not yet attended the program were not included in the statistics [24]. After 2010, a more than two times (from 3.3% in 2010 to 8.6% in 2016) increased HIV prevalence among PWIDs was observed in this study, which was not consistent with the notification of the decreased HIV transmission by IDU reported by China CDC [11]. However, the lack of NSP-related data made the cause of the trend obscure, and our findings implied that the promotion and effectiveness of NSP needs to be further evaluated.

Since 2005, HIV transmission due to IDU in China has been no longer the major transmission route, and the percentage of HIV infections due to IDU has decreased rapidly from 48.6% in 2005 to 3.8% in 2016 [11, 12]. However, our findings suggest that higher HIV prevalence among PWIDs, which increased over time (Table2, Fig. 1). Females, elder ages, minorities, the lower education, and the unmarried were associated with higher risk of HIV infection among PWIDs. In recent years, the proportion of female PWIDs infected with HIV continues to increase, and the HIV prevalence of other people getting infected through sexual transmission from female PWIDs is on the rise [25]. Condom promotion and mother-to-child prevention work have become the priority [26]. A prospective cohort study of 636 male PWIDs without HIV infection showed that lack of formal education was associated with loss of follow-up, which affected the rate of new HIV infections among PWIDs [27].

In our study, an intriguing finding was that individuals who received MMT before were more likely to be HIV infected was observed. Though it has been reported that MMT is associated with reduction in the risk of HIV infection among PWIDs, and syringe sharing behaviors in MMT groups diminished substantially [28, 29]. It needs to be emphasized that in terms of therapeutic effectiveness, greater benefit might be associated with longer duration of exposure to MMT and good adherence [28, 29]. However, in our study, these PWIDs who received MMT before entered detoxification centers, which showed the failure of MMT they received. And it might because they were more addicted to drugs, or the interference of other adverse factors. This phenomenon showed that the failure of MMT can pose a greater risk. Therefore, we need to focus not only on whether to receive MMT, but also on the effectiveness and outcome of the MMT. Key actions should be taken to strengthen the management of patients in MMT programs, and to improve the treatment adherence and success rate.

From a spatial perspective, our findings indicated that the HIV prevalence among PWIDs in southwest and northwest regions presented decreased trends, and both regions were traditional epidemic areas of opioids abuse with high risk of HIV infection [12]. Our results show that NSP programs might achieve some success in reduced HIV transmission in western regions, but the HIV prevalence among PWIDs in eastern region presented an increased trend. In 2009, Wei Guo’s study concluded that the proportion of PWIDs in central region maintained at about 50% among all drug users, and suggested that, once the number of HIV infections of drug users reached a high level, it was likely to cause a rapid increase in the HIV prevalence among drug users in this area [26]. Central and eastern regions in China contain population densities of > 450 people per square kilometer and account for about 46% of China’s population [30]. This finding implies that the government needs to become more aware of the risk of HIV transmission to the general population.

The HIV prevalence in our study could provide more valuable information on HIV epidemic than the number of HIV infections reported by China CDC. Yet, our study has several limitations. First, this is a series cross-sectional study rather than a cohort study, thus the HIV prevalence might contain bias. Second, the PWIDs sample undergoing detoxification in this study may have been injecting longer than other PWIDs, and therefore this study might have a higher HIV prevalence and may overestimate the prevalence among PWIDs. The last, the limited available data make it hard to draw conclusions what actually led to the increase in the HIV prevalence among PWIDs. To our knowledge, this is the first study of the HIV prevalence among the largest population of PWIDs nationwide in China. The unexpected findings of the U-shaped curve for the HIV prevalence among PWIDs suggest that current strategies on the HIV prevention and control among PWIDs might no longer be effective, and the reassessment is urgently needed.

## Conclusions

The U-shaped curve of the HIV prevalence among PWIDs implies that IDU might still be a critical transmission route of HIV infections in China. Besides the implementation of traditional model of HIV prevention, it is necessary to continue to strengthen the coverage and promotion of NSP and MMT, and pay attention to the quality of service, especially the management of MMT treatment adherence, and formulate a regular mechanism to evaluate effectiveness of comprehensive interventions. In addition to detoxification treatment, a sound social support network is needed with the community as the core. In our study, female, elder people, minority, lower education are exposed to HIV risk associated with IDU. Indicated prevention strategies should target these sub-populations. The western regions, which were traditional HIV high risk areas, the intervention of IDU is still the key point.

## Supplementary Information

**Additional file 1**. Univariate and multicariate analysis of HIV infection among PWIDs in China, 2008–2016.

**Additional file 2**. Trends in the prevalence of HIV infection among PWIDs by regions.

## Data Availability

The dataset of this study is available from the corresponding author on reasonable request.
