# Supplementary material for: Epidemic of HIV infection among persons who inject drugs in mainland China: a series, cross-sectional study
Source: Harm Reduct J. 2021 Jun 12;18:63. doi: 10.1186/s12954-021-00511-6 (PMC8199561; doi:10.1186/s12954-021-00511-6)
Supplement: Supplementary file 1 — Additional file 1. Univariate and multicariate analysis of HIV infection among PWIDs in China, 2008–2016. [file 12954_2021_511_MOESM1_ESM.docx]

**Additional file 1**

**Table S1. Univariate and multivariate analysis of HIV infection among PWIDs in China, 2008-2016**

|  | **OR(95%CI)** | **AOR(95%CI)^a^** | **AOR(95%CI)^b^** |
| --- | --- | --- | --- |
| Year |  |  |  |
| 2008 | 1.00 | 1.00 | 1.00 |
| 2009 | 0.87(0.77-0.98) ^*^ | 1.00(0.87-1.14) | 0.99(0.87-1.13) |
| 2010 | 0.77(0.69-0.86) ^***^ | 0.77(0.68-0.86) ^***^ | 0.75(0.67-0.85) ^***^ |
| 2011 | 1.12(0.98-1.28) | 1.07(0.93-1.23) | 1.05(0.92-1.21) |
| 2012 | 1.21(1.06-1.38) ^**^ | 1.05(0.91-1.20) | 1.04(0.91-1.19) |
| 2013 | 1.16(1.02-1.31) ^*^ | 1.02(0.90-1.16) | 1.02(0.90-1.15) |
| 2014 | 1.20(1.07-1.33) ^**^ | 1.19(1.07-1.33) ^**^ | 1.18(1.06-1.32) ^**^ |
| 2015 | 1.32(1.19-1.46) ^***^ | 1.21(1.09-1.34) ^***^ | 1.20(1.09-1.34) ^***^ |
| 2016 | 1.12(0.98-1.27) | 1.12(0.98-1.28) | 1.13(0.99-1.28) |

PWIDs: persons who inject drugs.

AOR of HIV test year all took the prior year as the reference.

AOR^a^ adjusted for sociodemographic characteristics, HIV test region and drug types.

AOR^b^ adjusted for sociodemographic characteristics, HIV test region, drug types and methadone treatment status.

*** suggested p<0.001; ** suggested p<0.01; ** suggested p<0.05
