# Supplementary material for: Epidemic of HIV infection among persons who inject drugs in mainland China: a series, cross-sectional study
Source: Harm Reduct J. 2021 Jun 12;18:63. doi: 10.1186/s12954-021-00511-6 (PMC8199561; doi:10.1186/s12954-021-00511-6)
Supplement: Supplementary file 2 — Additional file 2. Trends in the prevalence of HIV infection among PWIDs by regions. [file 12954_2021_511_MOESM2_ESM.docx]

**Additional file 2**


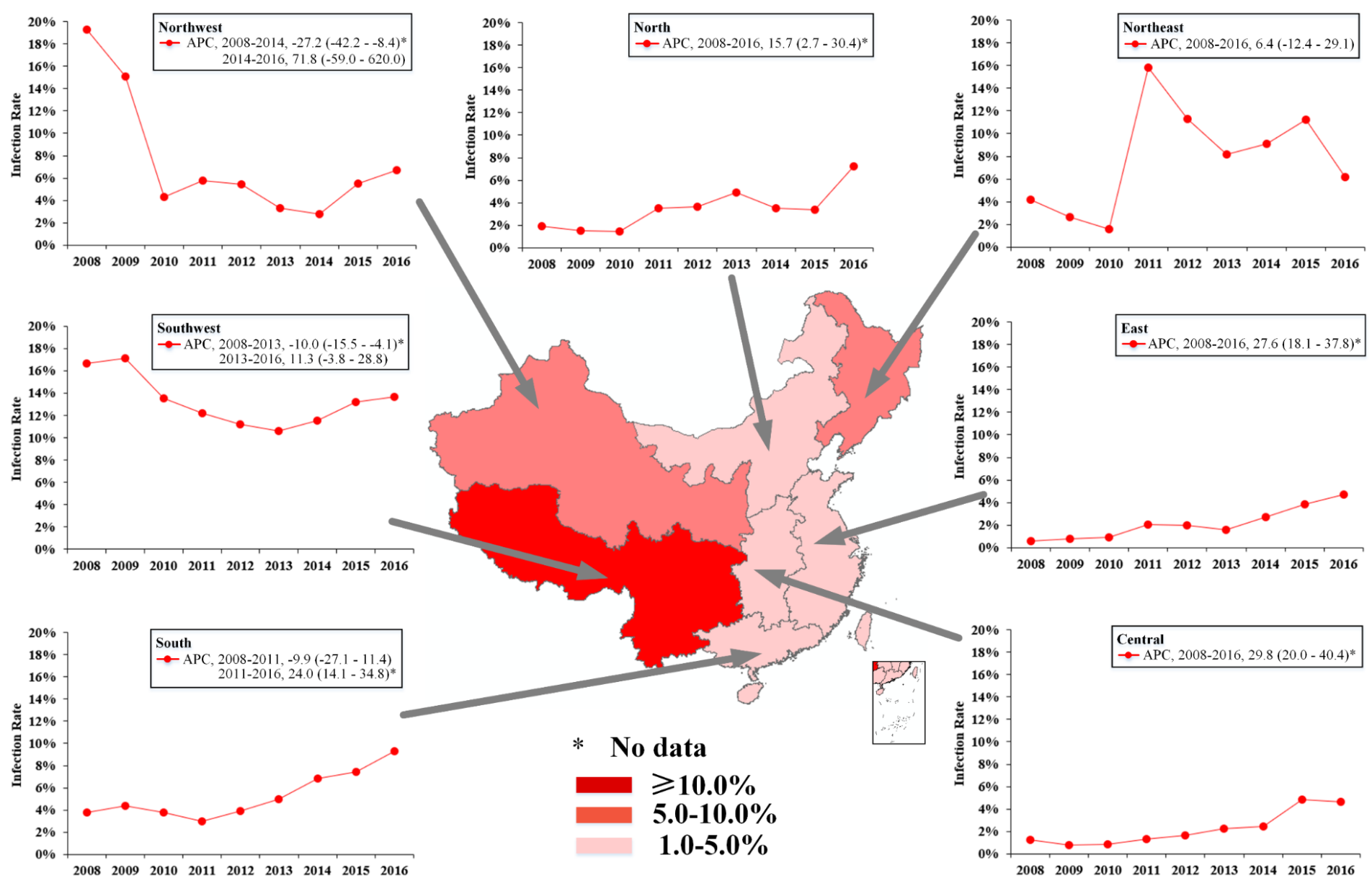


*suggested p<0.05

**Figure S1. Trends in the prevalence of HIV infection among PWIDs by regions**
